# A retrospective evaluation of fondaparinux for confirmed or suspected heparin-induced thrombocytopenia in left-ventricular-assist device patients

**DOI:** 10.1186/1749-8090-9-55

**Published:** 2014-03-21

**Authors:** Scott T Benken, Nicholas Tillman, Suhuir Dajani, Aesha Shah, Toby Thomas

**Affiliations:** 1 Department of Pharmacy, The University of Chicago Medicine, 5841 S. Maryland Ave. TE026, MC0010, Chicago, IL 60637, USA; 2 Department of Clinical Sciences, Roosevelt University College of Pharmacy, Schaumburg, IL, USA

**Keywords:** Left-ventricular-assist device, Heparin-induced thrombocytopenia, Thrombosis, Bleeding, Fondaparinux, Anticoagulation

## Abstract

**Background:**

Thrombotic events are a common complication of left ventricular assist device placement and warrant prophylactic anticoagulation. Heparin is the most common anticoagulant used for prophylaxis of thrombotic events in left ventricular assist device patients as a transition to oral anticoagulants but carries the risk of heparin-induced thrombocytopenia. Limited data is available for the treatment of heparin-induced thrombocytopenia in this patient population. We report an evaluation of 8 left ventricular assist device patients with suspected or confirmed HIT started on fondaparinux at the time of heparin-induced platelet-factor-4 antibody positivity.

**Methods:**

Adult patients were reported if they were heparin-induced platelet antibody positive, tested via enzyme-linked immunusorbent assay, post-operative after left-ventricular assist device, and were initiated on fondaparinux at the time of heparin-induced platelet antibody positivity. Waiver of informed consent was granted from the institutional review board. Baseline demographics, clinical course of HIT, safety and efficacy variables were collected.

**Results:**

Eight patients receiving fondaparinux were identified and included in this report. The patient group was on average 49 years old, weighing 95 kg, with calculated BMI 28.8 and consisted primarily of Caucasian males. Three patients developed new thromboses after initiation of fondaparinux for heparin-induced thrombocytopenia. Only one patient had a major bleeding event of an overt bleed after initiation of fondaparinux therapy.

**Conclusions:**

Given the lack of major bleeding in this evaluation, fondaparinux could be a potentially safe treatment option for left ventricular assist device patients that are heparin-induced platelet antibody positive pending confirmatory testing results. Given the development of new thromboses in 3 of 8 patients, concern exists about the efficacy of fondaparinux in this patient population. Significant limitations exist regarding these conclusions in this evaluation. Controlled, systematic evaluations are necessary to delineate safety and efficacy of fondaparinux for heparin-induced thrombocytopenia in this population.

## Background

The management of patients with heart failure has progressed in recent years due to the increased utilization of ventricular assist devices (VADs), including left ventricular assist devices (LVADs) [1,2]. According to the Centers for Disease Control and Prevention, an estimated 5.8 million people in the United States have heart failure and 550,000 are newly diagnosed with end stage heart failure each year [1]. Because of this burden, there has been an increased utilization of LVADs [2]. Data with contemporary LVAD’s (Heartware and Heartmate II) suggest an increased 1-year survival compared to historical data of medical therapy and it is estimated that > 3000 LVADs are implanted worldwide each year [1-5].

Although LVADs are potentially lifesaving and life extending, there are serious complications that arise post-operatively; most prominently bleeding and thrombosis [6,7]. Bleeding complications often lead to re-operation and occur more frequently than in other cardiac surgery and medical patient populations [8-10]. Thrombotic events in VAD patients occur at a reported incidence ranging up to 26% and because of this, all LVAD patients will be transitioned to long-term anticoagulation unless contraindicated [11]. Per consensus guidelines, it is recommended that LVAD patients be bridged with intravenous heparin to warfarin beginning as early as postoperative day 1 until therapeutic international normalized ratio (INR) is reached [12].

Given the common use of heparin, LVAD patients have an increased incidence of heparin-induced thrombocytopenia (HIT). The incidence approaches ~10%, which is twice as frequent as other post-surgical populations [13]. Due to the already high disease-related-rates of thromboses and significantly higher risk of bleeding complications at baseline, anticoagulation management for HIT in LVAD patients presents a clinical challenge.

Alternative anticoagulants that can been considered for the initial treatment of HIT patients, include selective factor Xa inhibitors (danaparoid and fondaparinux) and direct thrombin inhibitors (DTI’s: argatroban, lepirudin, desirudin, and bivalirudin) [14]. In the United States, the commonly utilized therapies for HIT include argatroban and bivalirudin (approved for HIT during percutaneous coronary intervention). Anecdotally, fondaparinux is utilized for this indication. One study found success while utilizing argatroban to manage HIT in VAD patients without an increased risk of bleeding [15]. Bivalirudin has been used during cardiac surgery in patients with a history of HIT, but its use in the treatment of HIT in LVAD patients has been shown only in case reports [16-18]. Fondaparinux has only one published case specifically in VAD patients (biventricular VAD only), no published cases with LVADs, but experience is growing in cardiac surgery patients [19-21].

Fondaparinux has pharmacologic characteristics that would make it desirable for the use of HIT in this population [22]. It is rapidly and completely absorbed after subcutaneous administration requiring no intravenous access. It also is distributed mainly in the blood which leads to predictable activity that should not be affected by changing volume of distribution in hospitalized patients. Further, it has peak anti-Xa activity occurring fairly rapidly in approximately 2–3 hours, requires no biotransformation for activity or elimination, and has no required monitoring parameters. Because of this, we investigated our institutional experience utilizing fondaparinux in LVAD patients with suspected or confirmed HIT.

## Methods

This was a retrospective, single-center, tertiary care, academic medical center evaluation. It was approved by the Northwestern Medicine Institutional Review Board (IRB) and due to the retrospective nature of the evaluation, waiver of informed consent was obtained. Adult patients (>18 years) who tested positive (optical density [OD > 0.4]) by enzyme-linked immunosorbent assay (ELISA) for the heparin-induced platelet antibody (HIPA) were identified and included. A query was done of these patients’ electronic medical records (EMR) from December 2008 to January 2012 for patients who received a LVAD (Heartware, Heartmate or Heartmate II). These lists were then cross-referenced to identify those LVAD patients who were HIPA positive and received fondaparinux during the evaluation period.

Major bleeding was defined as fatal bleeding, bleeding in a critical organ (retroperitoneal, intracranial, intraocular, etc.), deep hematoma, overt bleeding (an acute 2 g/dl or greater decrease in hemoglobin level), or bleeding leading to the transfusion of at least 2 consecutive units of packed red cells (PRBCs) within a 24 hour period. Thrombosis was detected by clinical suspicion coupled with standard diagnostic testing. For deep vein thrombosis, diagnosis required positive doppler ultrasonography; for pulmonary embolism, computed tomography was used to confirm diagnosis; for pump thrombosis, elevations of lactate dehydrogenase, decrease in haptoglobin, and surgical attending confirmation were required. Of note, surveillance diagnostic detection was not performed during the evaluation period. New thrombosis after anticoagulation was defined as the development of a new thrombosis after HIT diagnosis until 3-month follow-up. Isolated HIT was described as HIT with only platelet reduction at time of diagnosis [23]. Heparin induced thrombocytopenia and thrombotic syndrome was described as HIT associated with concomitant thrombosis [23]. Selection of fondaparinux for anticoagulation was determined by the treating providers at the time of treatment and given the retrospective nature of this evaluation, reasons for selection were not elucidated. It is important to note, that all Heartware patients were started on aspirin 325 mg daily and Heartmate II patients on aspirin 81 mg daily according to hospital protocol. Data is presented in medians and interquartile ranges (IQR) unless otherwise specified.

## Results

Eight patients were identified with a positive HIPA test in the post-operative setting status post LVAD implantation that received fondaparinux (Table 1). The patient group had a median age of 49 years old (7.8 y/o), weighing 95 kg (21.2 kg), with calculated BMI 28.8 kg/m^2^ (4.3 kg/m^2^) and consisted primarily of Caucasian (87.5%) males (75%). Only one (12.5%) patient had a history of VTE prior to admission, no patients had a history of diagnosed bleeding disorders, and two (25%) patients had a history of chronic kidney disease (CKD; defined as estimated GFR < 60 mL/min/1.73 m^2^ for ≥ 3 months). Six (75%) patients received Heartmate II devices and 2 (25%) received Heartware LVADs.

**Table 1:** HIT characteristics

**Patient**	**Type of LVAD**	**Admission Plt (K/uL)**	Temporal Plt nadir (K/uL)*	**ELISA OD**	4-T probability**	**Heme-onc consult**	SRA***	**HITTS**^**T**^	**Time of heparin exposure prior to LVAD surgery (days)**^**TT**^	**Time between heparin exposure and HIT Ab + (days)**
1	Heartmate II	157	127	0.54	Low	Y	(-)	No	12	19
2	Heartmate II	120	80	0.82	Intermed	N	n/a	No	n/a	0^t^
3	Heartware	203	99	0.44	Intermed	Y	n/a	DVT	17	21
4	Heartware	217	118	0.64	Low	N	(-)	No	6	10
5	Heartmate II	166	102	0.63	Low	N	n/a	No	n/a^TTT^	6
6	Heartmate II	218	30	0.45	Intermed	Y	n/a	No	9	8
7	Heartmate II	42	16	0.63	Low	Y	(-)	DVT, PE, stroke	13	1^t^
8	Heartmate II	526	45	3.15	High	Y	(+)	PE	5	5

*Temporal Plt Nadir = Plt count nearest (within 24 hr) of HIT antibody + .

**4-T probability: High-probability 6–8 points, intermediate probability 4–5 points, low probability ≤ 3 points.

***SRA is a send out laboratory assay at our institution with a typical turn-around time of 3–5 days.

^T^HITTS = diagnosed thrombosis at the time of HIT antibody + .

^TT^Data unavailable regarding preadmission or outside facility heparin exposure for any patient (eg. cardiac catheterization).

^TTT^First known exposure to heparin was intraoperative.

^t^Patient developed + HIT antibody on a subsequent admission after initial admission for LVAD surgery.

HIT = heparin-induced thrombocytopenia.

LVAD = left-ventricular-assist device.

Plt = platelet.

OD = optical density.

SRA = serotonin release assay.

HITTS = heparin-induced thrombotic and thrombocytopenic syndrome at time of HIT antibody + .

The admission median platelet count was 185 k/ul (70 k/ul) with a median decrease of 48.4% (30.7%) to a temporal (nearest platelet count within 24 hr to the HIT + antibody) platelet nadir of 57 k/ul (52 k/ul)*.* The HIT antibody was drawn 5 days (57.8 days) after admission to the intensive care unit (ICU) from the operating room. The 4-T pre-test probability of HIT demonstrated: 4 (50%) low probability, 3 (37.5%) intermediate probability, and 1 (12.5%) high probability [24]. The median HIPA OD via ELISA was 1.11 (0.63). Five of the eight (62.5%) patients received hematology consults to assist in the management of HIT. Confirmatory testing with the serotonin release assay (SRA) was sent on four (50%) patients (send-out laboratory test at our institution) and one returned as positive. Of note, the one patient with a positive SRA also had an ELISA OD of 3.15 and high probability on 4 T scoring.

At time of diagnosis, 3 (37.5%) patients had heparin-induced thrombotic-thrombocytopenic syndrome (HITTS) and 5 (62.5%) with isolated HIT (iHIT). The patients that presented with HITTS had the following thromboses: 1 patient – DVT alone, 1 patient – DVT, PE, and probable acute ischemic stroke, and 1 patient – PE alone. Patients were started on fondaparinux at the time of HIPA positivity at a median dose of 5 mg (Table 2). The median duration of fondaparinux treatment was 4.5 days (8 days) with 6 of 8 (75%) patients being transitioned to warfarin before discharge (2 patients discharged on fondaparinux). All patients discharged on warfarin were discharged with an INR within goal range of 2–3.

**Table 2:** Treatment characteristics

**Patient**	**CKD**	**Fondaparinux dose**	**Duration of fondaparinux (days)**	**Transitioned and discharged on warfarin (Y/N)**	**Concurrent aspirin dosage**	New thrombosis after fondaparinux*	**Major bleeding after fondaparinux**
1	Y	2.5 mg	5	Y	81 mg	No	No
2	N	2.5 mg	22	N	81 mg	LVAD thrombosis	No
3	N	5 mg	14	Y	325 mg	No	No
4	N	5 mg	4	Y	325 mg	No	No
5	N	5 mg	3	Y	81 mg	PE	No
6	N	7.5 mg	10	Y	81 mg	DVT	Overt bleed**
7	N	7.5 mg	3	N	81 mg	No	Blood transfusion***
8	Y	10 mg	2	Y	81 mg	No	Blood transfusion***

*All thrombotic events occurred prior to discharge on the admission in which HIT was diagnosed.

**Overt bleeding = ≥ 2 gram g/dL decrease in any 24 hr period.

***Believed to be related to clinical course and unrelated to anticoagulation therapy.

CKD = chronic kidney disease; defined as estimated glomerular filtration rate (GFR) < 60 mL/min/1.73 m2 for > 3 months.

DVT = deep vein thrombosis.

PE = pulmonary embolism.

Three (37.5%) patients developed a new thrombotic event after the diagnosis of HIT despite the initiation of fondaparinux transitioned to therapeutic warfarin prior to discharge (1 patient – PE, 1 patient – LVAD thrombosis, 1 patient – DVT). It was not routine practice during the evaluation period to perform surveillance diagnostic testing unless clinical suspicion was raised and thus thromboses were only diagnosed after clinical concern developed. All patients that developed a new thrombotic event while receiving fondaparinux presented with iHIT at time of HIT diagnosis. There were no bleeding events into any critical organs and no fatal bleeding. One overt bleeding event was reported, but did not warrant cessation of anticoagulation or red blood cell transfusion. Two (25%) patients required red blood cell transfusion after the initiation of fondaparinux though the need for transfusion was not believed to be related to anticoagulation therapy. In the two patients that required transfusion, the admission hemoglobin (Hgb) was 12.4 and 15 mg/dL with a nadir Hgb 6.7 and 8.6 mg/dL and average Hgb throughout admission of 9.4 and 10.2 mg/dL, respectively. Each patient only required one transfusion. No patients died or were discharged to hospice and all patients were successfully discharged home or to rehabilitation.

## Discussion

In our evaluation we experienced only one major bleeding episode with the use of fondaparinux for confirmed or suspected HIT. Given the lack of major bleeding complications, it appears that fondaparinux could be a safe option for the treatment of HIT in LVAD patients. It is of special importance to find safe alternative anticoagulants for the treatment of HIT in a patient population that is already predisposed to bleeding. Post-cardiac surgery patients are at an increased risk of bleeding due to both surgical and non-surgical causes. Approximately 20% of post-cardiac surgery patients bleed significantly due to surgical bleeding [25]. These patients are also at risk for bleeding because of undergoing cardiopulmonary bypass (CPB). Cardiopulmonary bypass can increase bleeding due to exposure of procedural high dose anticoagulation (eg. heparin), extensive surgical trauma, prolonged exposure of blood to an artificial surface leading to reactant activation of thrombolysis and fibrinolysis, as well as significant platelet dysfunction from a CPB-induced loss of receptors for von Willebrand factor (GP1b) and fibrinogen (GP2b3a) [26]. Beyond that, patients with continuous flow LVAD have been shown to develop a relative von Willebrand factor deficiency which is hypothesized to contribute to the bleeding incidences in this population [27]. Because we observed minimal bleeding in light of these risks, this agent may prove to be safe for this patient population.

There is concern for efficacy in this small sample size due to 3 of the 8 patients developing new thrombotic events following initiation of fondaparinux after HIT diagnosis. Data with FDA approved DTI’s, (lepirudin and argatroban) have demonstrated new thromboses from HIT after initiation of alternative anticoagulation at lower rates in other patient populations (6-10% and 13-19% of patients respectively) [28-34]. Comparison between this historical data and our evaluation must be made with caution because the post-cardiac surgery LVAD patient population carries a significant risk of clotting that others may not. It has been demonstrated that patients requiring CPB have a high expression of tissue factor (TF) and activated factor VII (fVIIa) due to direct contact with the CPB-circuit [27,35]. This expression leads to the activation of the contact coagulation pathway as well as activation of the intrinsic coagulation pathway leading to thrombin generation. Beyond that, patients that undergo CPB develop systemic inflammation [27]. Through acute-phase inflammatory reactants such as leukocytes, interleukin 1, tumor necrosis factor, and endotoxin, TF expression is increased on the endothelium leading to further activation of the coagulation pathways [27]. Further, LVAD patients are known to be at a high risk for thrombosis. Recent evaluation has shown fibrin deposition as a possible culprit leading to hemolysis, increase in shear stress, and pump thrombosis [36]. Because of these factors, comparisons with historical data are confounded because significant thrombotic risks exist in the post-cardiopulmonary bypass LVAD population.

Because fondaparinux has not been systematically studied in this population for this indication, it is unknown if the thrombotic observations noted were dose related and if efficacy could be optimized using different dosing strategies. It is important to note, that based on current treatment recommendations for acute DVT/PE, only 3 of the 8 patients evaluated received appropriate weight-based doses [22]. Of the three patients that developed thrombosis after initiation of fondaparinux, only 1 of them was on the appropriate weight-based dose (data not shown). There is recommendation for dose reduction in renal dysfunction, but renal function data was not collected and thus appropriateness of dosing cannot be fully evaluated. Because post-operative LVAD patients often develop varying degrees of renal insufficiency, there is likelihood that dose modification based on renal function was present. Without this information, this is only speculation. Further, during the observation period and to date there are not recommendations for routine utilization of anti-Xa serum concentrations to assess fondaparinux efficacy. If this information were available in our analysis, firmer conclusions could be made regarding the efficacy of the current doses in relation to our thrombotic observations. Also, given the retrospective nature of this evaluation, it is difficult to determine if thrombotic variables we observed were related to HIT, its treatment, or to the clinical course of LVAD therapy.

Confounding our observations further, it appears that in our evaluation there is potential for a high number of false positive HIPA tests. Because the 4-T pre-test probability demonstrated mostly low and intermediate probability with only one patient with high probability and only one patient ELISA OD value greater than 1, it is possible we were treating false-positive HIT antibodies [24,29]. It has been shown that patients with an ELISA OD less than 1 only had a 3.4% positive diagnosis for HIT, an OD 1–2 demonstrated 45.5% positive diagnosis, and greater than 2 with 78% positive diagnosis [37]. Of note, the 4-T pre-test probability was calculated retrospectively and thus this information was not explicitly found in the medical record or stated as available for the treating clinicians. It has been found previously that there are a large number of false positive HIT antibodies in post-cardiac surgery patients and that often a low platelet count remains so for non-HIT reasons [38-40]. These patients develop a consumptive thrombocytopenia from cardiopulmonary bypass required during LVAD implantation which also has been shown to release platelet factor 4 which could lead to false positivity with ELISA testing [41]. It is also important to point out that only 50% of the patients in our analysis had confirmatory testing sent (Table 1) and without confirmatory testing, it is difficult to know if we are treating true HIT.

There are numerous characteristics which would make fondaparinux an attractive agent in this high-bleeding/high-thrombotic risk population. First, it has a lower burden of therapy. One could hypothesize that treatment with fondaparinux is easier than continuous infusion DTI’s given it is administered once daily with minimal required monitoring parameters and does not require intravenous access. Also, fondaparinux potentially allows for an easier transition to warfarin than argatroban as it has no significant effect on the INR which is desirable as LVAD patients have indications for warfarin therapy beyond HIT itself [41]. Because it is distributed in the blood, changes that occur in LVAD patients’ volume status post-operatively are not likely to affect the pharmacokinetics and pharmacodynamics of this medication. Also, it does not need or undergo hepatic biotransformation which may allow for utilization in patients that would otherwise be challenging with argatroban. Though it does require dose modification for renal dysfunction, more data is emerging on how to optimize this medication in this patient subset [41]. Lastly, in terms of safety, the major bleeding rates of fondaparinux when utilized for HIT appear very low especially in comparison to the bleeding rates with DTI’s which would be of potential benefit in a patient group at high risk for bleeding complications such as LVAD patients, but without head-to-head comparator trials, this conclusion cannot be confirmed [19,31-33]. All of these attractive characteristics would have to be balanced with the potential concerns for efficacy seen in our observations but prospective or large retrospective comparator trials would be warranted to attempt to elucidate this information.

## Conclusions

Given the low incidence of major bleeding complications with therapy, fondaparinux may be a useful agent in a high-bleeding-risk population, such as LVAD patients, for the treatment of HIT. Based on our observations, concern for efficacy exists, especially in this highly thrombogenic patient population. Prospective, multi-center or large registry comparator evaluations are necessary to further investigate this question.

## Abbreviations

VAD: Ventricular assist device; LVAD: Left ventricular assist device; INR: International normalized ratio; HIT: Heparin-induced thrombocytopenia; DTI: Direct thrombin inhibitor; OD: Optical density; ELISA: Enzyme-linked immunosorbent assay; HIPA: Heparin-induced platelet antibody; EMR: Electronic medical record; PRBC: Packed red blood cell; iHIT: Isolated heparin-induced thrombocytopenia; HITTS: Heparin-induced thrombocytopenia and thrombotic syndrome; IQR: Interquartile range; CKD: Chronic kidney disease; GFR: Glomerular filtration rate; ICU: Intensive care unit; SRA: Serotonin release assay; DVT: Deep vein thrombosis; PE: Pulmonary embolism; Hgb: Hemoglobin; CPB: Cardiopulmonary bypass; FDA: Food and drug administration; TF: Tissue factor; fVIIa: Activated factor seven.

## Competing interests

The authors declare that they have no competing interests.

## Authors’ contributions

SB conceived the investigation, was responsible for evaluation coordination, IRB submission, over-seeing data collection, and manuscript composition. SD, NT, AS and TT participated in evaluation strategy, data collection, and manuscript composition. All authors read and approved the final manuscript.
